# Sea-ice retreat suggests re-organization of water mass transformation in the Nordic and Barents Seas

**DOI:** 10.1038/s41467-021-27641-6

**Published:** 2022-01-10

**Authors:** G. W. K. Moore, K. Våge, I. A. Renfrew, R. S. Pickart

**Affiliations:** 1 Department of Physics, University of Toronto, Toronto, ON Canada; 2 Department of Chemical and Physical Sciences, University of Toronto Mississauga, Mississauga, ON Canada; 3 Geophysical Institute, University of Bergen, Bergen, Norway; 4 Bjerknes Centre for Climate Research, Bergen, Norway; 5 School of Environmental Sciences, University of East Anglia, Norwich, UK; 6 Woods Hole Oceanographic Institution, Woods Hole, MA USA

**Keywords:** Physical oceanography, Atmospheric dynamics, Cryospheric science

## Abstract

Water mass transformation in the Nordic and Barents Seas, triggered by air-sea heat fluxes, is an integral component of the Atlantic Meridional Overturning Circulation (AMOC). These regions are undergoing rapid warming, associated with a retreat in ice cover. Here we present an analysis covering 1950−2020 of the spatiotemporal variability of the air-sea heat fluxes along the region’s boundary currents, where water mass transformation impacts are large. We find there is an increase in the air-sea heat fluxes along these currents that is a function of the currents’ orientation relative to the axis of sea-ice change suggesting enhanced water mass transformation is occurring. Previous work has shown a reduction in heat fluxes in the interior of the Nordic Seas. As a result, a reorganization seems to be underway in where water mass transformation occurs, that needs to be considered when ascertaining how the AMOC will respond to a warming climate.

## Abstract (web summary)

Water mass transformation in the Nordic and Barents Seas is important for the Atlantic Meridional Overturning Circulation (AMOC). Here, the authors show increases in air-sea heat fluxes linked to sea ice retreat along the boundary currents of the Nordic and Barents Seas that could influence how the AMOC reacts to climate change.

## Introduction

The northward transport of warm and salty water within the North Atlantic Ocean, emanating from the Gulf Stream system, plays a fundamental role in the Earth’s climate^1,2^. The wintertime densification of this Atlantic Water, via the transfer of heat and moisture to the atmosphere as it passes through the Nordic (Norwegian, Greenland and Iceland) and Barents Seas, is an important contributor to the deep southward return flow of the AMOC^1,3–5^. This water mass modification produces Atlantic-origin overflow water along the rim current system encircling the Nordic Seas^4^. Atlantic Water also enters the Arctic Ocean through the east side of Fram Strait and the Barents Sea^6,7^ where it is further transformed^8^, impacting the thermohaline structure of the Arctic Ocean, as well as the distribution of sea ice^9^. Ultimately this modified Atlantic Water reenters the Nordic Seas through the west side of Fram Strait where it contributes to overflow waters crossing the Greenland-Scotland Ridge^4,10–12^. In addition, colder and fresher Arctic-origin overflow water is formed within the interior basins of the western Nordic Seas^11,13,14^. The regional air−sea interaction that is necessary for these water mass changes also impacts the atmosphere, through a warming and moistening of the atmospheric boundary layer^15^, as well as marine ecosystems^16^.

**Fig. 1:**
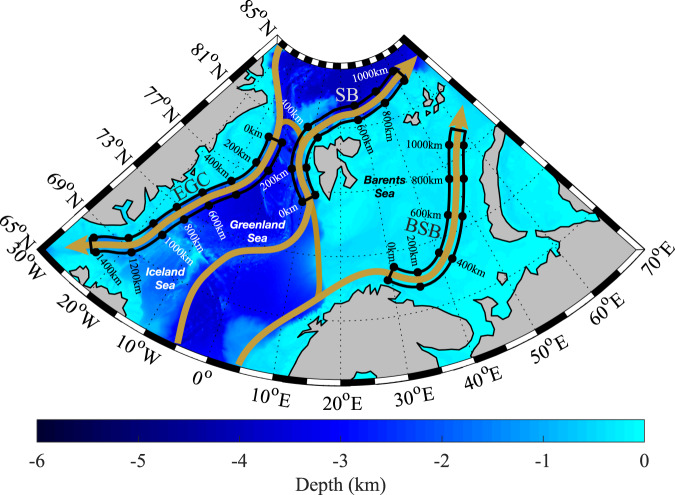
The bathymetry of the Nordic and Barents Seas. The domains associated with the East Greenland Current (EGC), the Svalbard Branch (SB), and the Barents Sea Branch (BSB) are shown in black with distances along the direction of the current flow indicated.

Within the Nordic and Barents Seas, there exist three major boundary currents where water mass modification occurs (Fig. 1): the East Greenland Current (EGC) that flows southward along the East Greenland shelfbreak/upper-slope from Fram Strait to Denmark Strait^17,18^; the Norwegian Atlantic Current that flows northward through Fram Strait into the Nansen Basin as the Svalbard Branch (SB)^7,8^; and the Barents Sea Branch (BSB) that progresses from the Norwegian Sea through the Barents Sea towards Novaya Zemlya^6,19,20^. These latter two branches merge in the Arctic Ocean to form the circumpolar Atlantic Water Boundary Current. Water mass modification also occurs throughout the central Nordic Seas within the Norwegian Sea’s Lofoten Basin^21^, as well as within the Iceland and Greenland Seas^3,10,13^. Both the EGC and SB flow along the shelfbreak and we used this characteristic to define the orientation of the domains for these currents (see Fig. 1); for the BSB the domain was defined based on the representation of its spatial extent^22^. For all three currents, we assume a width of 100 km (results were not sensitive to this width).

In the Nordic and Barents Seas, there is evidence of multi-decadal variability in winter ice cover^23^ that includes a significant expansion off East Greenland during the late 1960s and early 1970s, the so-called ice years^24^. More recently, there has been a sustained retreat of sea ice that has resulted in the disappearance of the Greenland Sea’s Odden Ice Tongue^25^, as well as reductions in ice cover across the northern and eastern parts of the Barents Sea^26^ and to the north of Svalbard^27^.

This retreat appears to be resulting in a reduction in air−sea interaction over the central Iceland and Greenland Seas that may be lessening the production of dense overflow waters there^10,28^. At the same time, this sea-ice retreat is exposing part of the EGC to the atmosphere, leading to enhanced air−sea interaction in that region^29^. Within the Barents Sea, the retreat of winter sea ice has resulted in profound changes in the climate—an Atlantification of the region^30^.

In this paper, we focus on the surface turbulent heat flux, which is the sum of the surface sensible and latent heat fluxes, with the convention being that fluxes out of the ocean are positive. This flux plays a dominant role in the high-latitude water mass transformation^3,31,32^. Please see the “Methods” section for additional details. Here we show that there is an increase in the air−sea heat fluxes along these currents that is a function of the currents’ orientation relative to the axis of sea-ice change suggesting enhanced water mass transformation is occurring

## Results

### Spatiotemporal variability in the air−sea heat fluxes and sea-ice

**Fig. 2:**
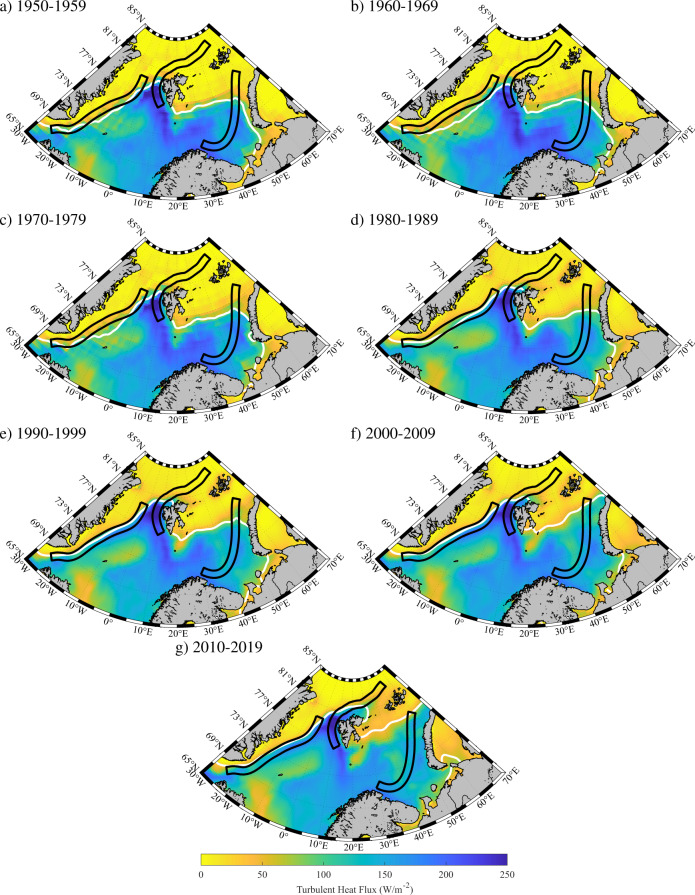
Spatiotemporal variability in winter mean turbulent heat flux over the Nordic and Barents Seas. Decadal means, **a**–**g**, are shown for the period 1950−2020. The domains associated with the East Greenland Current (EGC), the Svalbard Branch (SB), and the Barents Sea Branch (BSB) are shown in black. The 50% sea ice concentration contour is shown in white. All fields are from the ERA5 Reanalysis.

Figure 2 shows the spatiotemporal variability in the winter mean surface turbulent heat flux and sea-ice extent for decadal means between 1950 and 2020. The winter mean turbulent heat flux is typically small over ice covered regions, as a result of the insulating properties of sea ice, and increases rapidly across the marginal ice zones with maxima in the northern Greenland Sea, in the vicinity of Svalbard, as well as in the Barents Sea^10,28,33^. There are also minima in the Iceland and Greenland Seas that are the result of the competing influences of the two climatological low-pressure systems, the Icelandic and Lofoten Lows, that are prevalent in the region during winter^33^.

The variability in ice cover clearly modulates the winter-mean turbulent heat flux along the EGC, the SB, and the BSB. For example, in the Greenland Sea, an expansion of ice cover during the 1960s and its subsequent retreat over the following decades corresponds with a movement of the region of enhanced heat fluxes relative to the EGC, which is dynamically tied to the shelfbreak and upper slope along east Greenland^17^. Over both the Barents Sea and the region north of Svalbard, the retreat of sea ice has resulted in changes in the turbulent heat fluxes along the SB and the BSB currents.

### Along-current variability in the air−sea heat fluxes and sea-ice

**Fig. 3:**
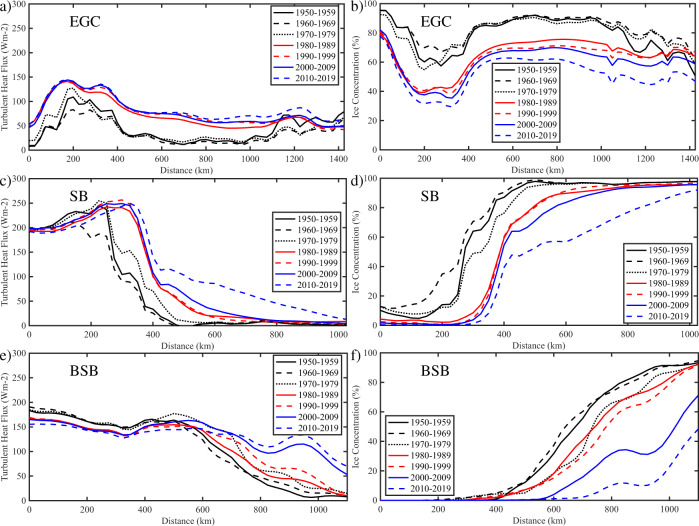
Evolution of the along-current winter mean turbulent heat flux and ice concentration. Decadal means, **a**, **c**, and **e** for the turbulent heat flux  and **b**, **d** and **f** for the ice concentration, are shown for the period 1950−2020. Results are shown for the East Greenland Current (EGC), the Svalbard Branch (SB), and the Barents Sea Branch (BSB). All fields are from the ERA5 Reanalysis.

To quantify these changes, decadal means of the along-current turbulent heat flux and sea ice concentration were constructed (Fig. 3). Over the EGC (Fig. 3a), there was a pronounced jump in the magnitude of the turbulent heat fluxes between the 1970s and 1980s. This was most noticeable along the middle of the EGC, from approximately 400 to 1100 km, where the increase was relatively uniform due to the retreat of sea ice being perpendicular to the current (c.f. Fig. 2). Prior to this transition, the turbulent heat fluxes were low, reaching a minimum during the ice years of the 1960/1970s. After this breakpoint, the fluxes were considerably larger along this centre section and showed only small changes with time. The situation at the northern and southern ends of the EGC was more complicated. At the northern end (i.e., small distances along the current), there was large variability in heat fluxes in the 1950s to 1970s, due to associated variability in sea-ice concentration (Fig. 3b). After this period, a significant retreat in sea-ice has limited the variability from the 1980s−2010s (Fig. 3b). At the southern end (i.e., large distances along the current), there has been high variability in heat fluxes and sea ice across the entire period.

The character of the turbulent heat flux along the SB (Fig. 3c) and BSB (Fig. 3e) are considerably different. Along both currents, the distance subject to high heat fluxes has increased through the decades. For the SB, the flux increase was largest between 250 and 600 km along the current, while for the BSB, the flux increase was largest between 600 and 1100 km. For both currents, this was due to a retreat of sea-ice cover, primarily in the same direction as the currents (Fig. 3d, f). There were differences in the timing of the largest changes in the heat fluxes. For the SB, this occurred during the 2000s; while for the BSB, the changes have been more uniform over time. At the southern end of both currents, there was a trend towards lower heat fluxes which was the result of a warmer and moister atmosphere there^28^.

Sea-ice retreat is instrumental to the changes in surface heat fluxes along all three currents. However, it is clear that the orientation of the current relative to the direction of sea-ice retreat is crucial in how the changes are manifested. For the EGC, the orientation of the current is approximately perpendicular to the direction of sea-ice retreat leading to changes in heat fluxes along the length of the current, while for the SB and BSB the currents are aligned with the direction of sea-ice retreat leading to changes in heat fluxes in focused regions.

### Along-current changes to the air−sea heat fluxes

**Fig. 4:**
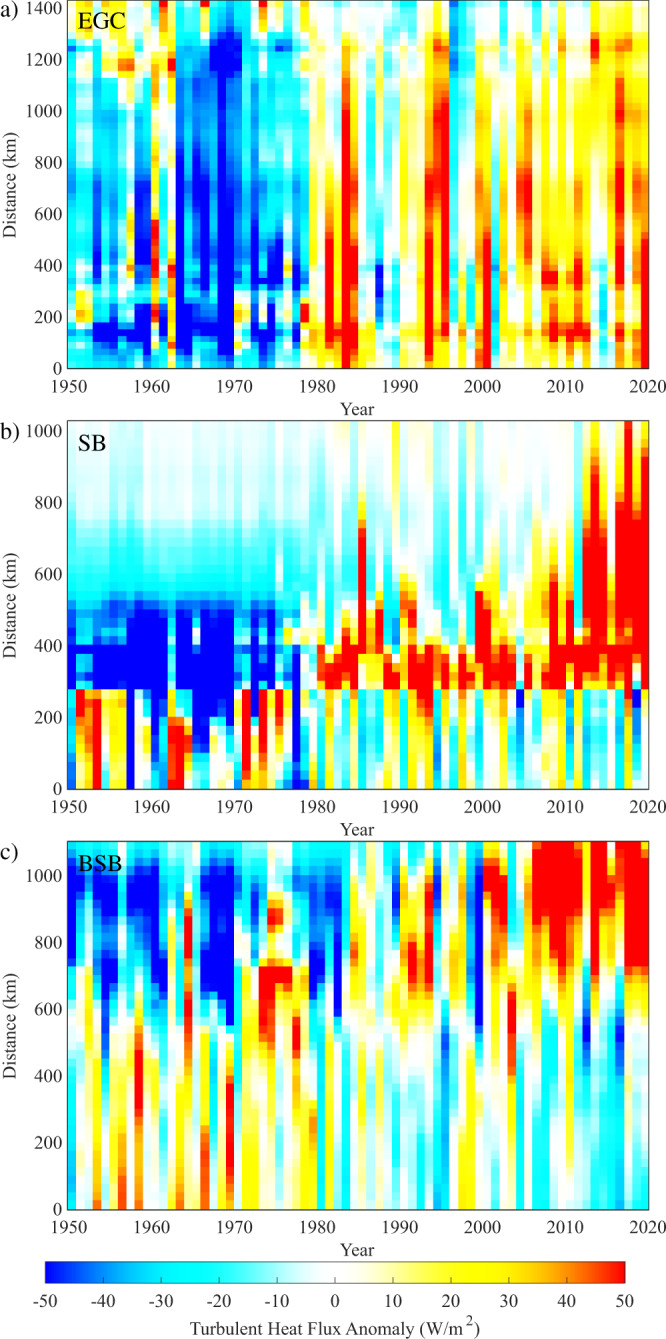
Hovmöller plots illustrating turbulent heat flux anomaly along the current domains. Panels are for the: **a** East Greenland Current (EGC); **b** Svalbard Branch (SB) and **c** Barents Sea Branch (BSB). The anomaly is defined as the difference with respect to the mean from 1950 to 2020. All fields are from the ERA5 Reanalysis.

Although the decadal means shown in Figs. 2 and 3 confirm that there is significant spatiotemporal variability in the heat fluxes along the three current systems, there is an arbitrariness to the decadal timescale used. A more flexible representation of this variability comes from Hovmöller plots of the turbulent heat flux anomaly (Fig. 4). For the EGC (Fig. 4a), one can see the large change in the heat flux that occurred after the ice years of the late 1960s and early 1970s and the characteristic that interannual variability often affects the entire length of the EGC. Along the SB (Fig. 4b), the evolution of the heat flux anomaly was quite different. Prior to the 1980s, the anomalies had a dipolar structure with positive anomalies to the west of Svalbard (i.e., distances less than 300 km) and negative anomalies to the north. After the 1980s, the sign of the dipole reversed with the region of positive values extending northeastward in recent years. A dipolar structure is also present along the BSB (Fig. 4c) with again a reversal in the sign of the anomalies around 1980.

The results presented so far indicate that there is complexity in the distribution of the heat fluxes along the three current systems (Fig. 3 and Supplementary Fig. 1). As noted, the differing orientation of the current systems relative to the direction along which the changes in sea ice were largest is critical. For the EGC, the step-function-like change in the heat fluxes along the entire current is the result of its orientation being normal to the direction along which ice has advanced then retreated. In contrast, for both the SB and BSB the striking reversals in sign of the heat flux anomalies are the result of the sea-ice generally retreating in the direction of the currents.

### Temporal variability of the along-current integrated air−sea heat fluxes

**Fig. 5:**
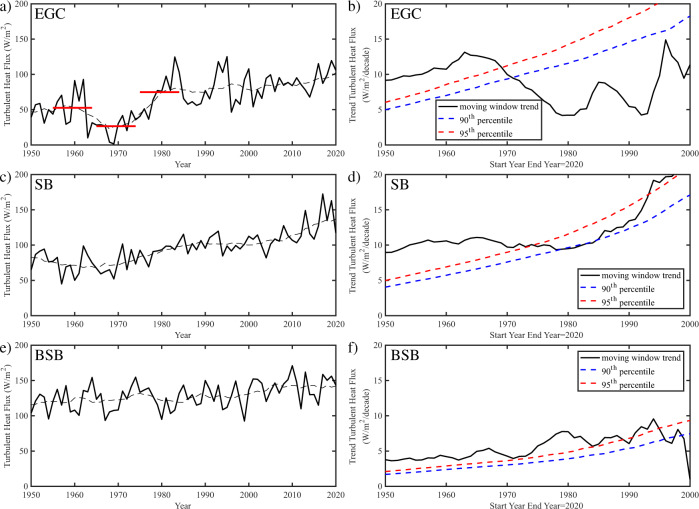
Temporal variability in along-current averaged winter mean turbulent heat flux. Time series of the turbulent heat flux are shown in the black curves for the: **a** East Greenland Current (EGC); **b** Svalbard Branch (SB) and **e** Barents Sea Branch (BSB) with the corresponding black dashed curves representing the low frequency variability as expressed by a 10-year moving window average. In **a** the decadal means over the period before (1955−1964), during (1965−1974) and after (1975−1984) the ice years are shown by the red lines. The trend in the winter turbulent heat flux are shown in the black curves as a function of a variable start year with a fixed end year of 2020 for the: **b** EGC, **d** SB and **f** BSB with the corresponding blue and red dashed curves representing the 90th and 95th percentile trends from a Monte Carlo distribution of time series that share the same spectral characteristics as the underlying time series. Trends with magnitudes larger than a given percentile curve are statistically significant at that threshold. All fields are from the ERA5 Reanalysis.

The along-current integrated heat flux, which quantifies the heat relinquished to the atmosphere, is a critical factor in the transformation of the water masses advected by the currents. Figure 5 shows the time series of the winter-mean heat flux averaged along the three current systems, as well as moving window trends with a variable start date and a fixed end date of 2020. The statistical significance of the trends is assessed using a Monte Carlo technique that uses 10,000 synthetic time series generated so as to retain the spectral characteristics of the underlying time series, thereby retaining any temporal autocorrelation that may reduce the degrees of freedom^28^.

For the EGC (Fig. 5a), the minimum in heat fluxes during the 1960s is evident, as well as the step-function-like increase in heat fluxes that occurred after this minimum. Indeed, the decadal mean for the ice years period 1965−1974 was 26 W/m^2^, half the 52 W/m^2^ of the preceding 10-year period (1955−1964) and only a third of the 75 W/m^2^ of the following 10-year period (1974−1984). The changes across each of these periods are statistically significant at the 95th percentile confidence level. The maxima in heat fluxes during the mid 1980s and 1990s stand out as well. The moving window trend for the EGC (Fig. 5b) increases for start dates up to the mid 1960s reaching a maximum of ~12 W/m^2^/decade that reflects the low heat fluxes during the ice years. After this time, the trend in the EGC winter mean heat flux is reduced by approximately 50% and is no longer statistically significant at the 95th percentile confidence level. The magnitude of the trend increases attaining values above 10 W/m^2^/decade for start dates after 1990 but they do not reach statistical significance. Over the period from 1950 to 2020, the winter mean heat flux averaged along the EGC increased from ~50 to ~100 W/m^2^, statistically significant at the 99th percentile confidence interval.

For the SB (Fig. 5c), the winter mean heat fluxes underwent a sustained increase after the 1960s that accelerated after 2000. The moving window trend of ~10 W/m^2^/decade during much of this period is statistically significant and increases to ~20 W/m^2^/decade for start dates after the mid 1990s, when it is also statistically significant, see (Fig. 5d). Over the period from 1950 to 2020, the winter mean heat flux averaged along the SB increased from ~75 to ~130 W/m^2^, statistically significant at the 99th percentile confidence interval.

For the BSB (Fig. 5e), there is significant inter-annual variability throughout the period, but no evidence of the decadal variability seen in the EGC or SB. Rather, there was a sustained increase in the winter mean heat flux averaged along this current from ~120 W/m^2^ during the 1950s to ~140 W/m^2^ during the 2010s. This relatively modest increase in integrated flux is because of the dipolar nature (Fig. 4c) of the anomalies along the current. This is confirmed by a moving window trend for this time series of 4−10 W/m^2^/decade for start dates from the 1950s−1990s (Fig. 5f).

## Discussion

These results confirm that the relative orientation of sea-ice retreat versus the current axis is critical for characterizing the changes in the surface heat fluxes. In particular, the large increase observed along the EGC is due to this current being perpendicular to the axis of sea ice retreat and therefore the entire current system is being exposed to the atmosphere at once. By contrast, for the SB and BSB the currents are parallel to the axis of sea-ice retreat resulting in dipolar changes along their length that reduces the magnitude of integrated heat flux.

The trends in turbulent heat flux along these three boundary currents are primarily a response to sea-ice retreat, although an element of differential warming between the atmosphere and ocean is also contributing (Supplementary Fig. 2). We have examined trends in the inflow of warm Atlantic water into the Nordic Seas using observations^34^ and find these are dwarfed by the trends in atmospheric temperature (Supplementary Fig. 3). The surface wind speed also plays a role in air−sea heat fluxes; however, the changes in wind speed over the Nordic Seas have been small^28^. The changing nature of water mass transformation in the Greenland and Iceland Sea gyres has been previously examined^28^. However, as that was a diagnostic study based on reanalysis data, they could not determine which mechanism was dominant. A recent analysis of control and global warming coupled model simulations for the region has found that sea-ice retreat dominates the surface heat flux change, although this is modulated by an increase in the northward transport of warm water^35^.

If sea-ice retreat is the dominant factor, then the recent changes experienced by these boundary currents will follow the evolution of the wintertime sea ice distribution. Once the wintertime sea ice has permanently retreated from a region, the differential warming of the atmosphere and ocean will lead to a decrease in the air−sea heat fluxes—as already evident along the southern sections of SB and BSB (Figs. 3, 4). Indeed, we would anticipate these reduced fluxes will extend northward over time. However, a key point is that once the boundary current is ice-free in winter, water mass transformation may occur directly within the current—if the surface fluxes are sufficiently strong such as occurs during cold air outbreaks in other regions, e.g., the Irminger and Labrador Seas^31,36^.

Previous work^28^ has shown that a trend towards lower heat fluxes within the Greenland and Iceland Sea gyres is leading to shallower oceanic mixed-layers and diminished volumes of overturned waters in the interior. This has the potential to reduce the ventilation of intermediate waters and the supply of dense overflow waters to the North Atlantic. In contrast, we have shown here that sea-ice retreat and the associated increase in heat fluxes along the boundary currents of this region has the potential to increase ventilation of intermediate waters and densify the Atlantic-origin overflow water being transported by these currents, as recently observed along the southern EGC^29^ and the SB^8^.

Since waters transformed within the boundary currents are readily transported to the overflows out of the Nordic Seas, this shift in the location of water mass transformation from the interior to the boundary has potentially profound implications for the lower limb of the AMOC—keeping in mind that a large contribution to the AMOC comes from the transformation occurring in the Nordic Seas^32^. Recent work^37,38^ indicates a slowdown of the AMOC over the last century. However, the enhanced boundary current transformation implicated in this study has the potential to impart resilience to the overturning north of the Greenland-Scotland Ridge which may help maintain the AMOC in a warming climate. Increased densification of this water should in fact lead to enhanced entrainment of ambient fluid as the dense water spills over the ridge^39^, which means more sinking. Further work is clearly required to document the large-scale impacts of wintertime ice retreat from the continental margins of the Nordic and Barents Seas.

## Methods

The documented variability in ice cover motivates an examination of the spatiotemporal variability of air−sea heat fluxes along these boundary currents. To accomplish this, the 5th generation reanalysis from the European Centre for Medium-Range Forecasts (ECMWF) known as ERA5 was used^40^. Both ERA5 and the well-established 4th generation reanalysis known as ERA-Interim^41^ are based on ECMWF’s Integrated Forecast System (IFS). The ERA5 data have a spatial resolution of ~30 km and cover the period 1950-onwards, which includes the recently released early-period extension. The sea surface temperature in ERA5 is a prescribed field that is a blend of a number of products from the UK Met Office and others^40^. In ice-covered regions, where observations are limited, the sea surface temperature is a regressed function of the sea ice concentration^42^.

It should be noted that the present study neglects the impact of precipitation and evaporation on the surface salinity (although evaporation is considered as it relates to the latent heat flux). This is because a number of studies have indicated that evaporation and precipitation over the region of interest are, on seasonal timescales, in balance^43,44^ resulting in only small changes to the surface density^31,45^. Freshwater fluxes from terrestrial sources are also neglected. While this could have relevance for the EGC due to its proximity to Greenland’s melting ice sheet^46^, it has been suggested^29^ that there is substantial Ekman transport associated with the strong northerly barrier flow during the fall and winter along the East Greenland coast that keeps this freshwater on the shelf, thereby limiting its impact on the EGC. A recent modelling study confirmed this scenario^47^. We also neglect a number of oceanographic and cryospheric processes that can have impacts on water mass modification along these currents^5,48^.

A comparison with in situ observations indicates that IFS-based reanalyses are able to represent the air−sea fluxes in these subpolar seas with a good degree of fidelity^49–51^. The complex spatial heterogeneity of the marginal ice zone leads to mesoscale variability^52^ that is not fully captured in reanalysis datasets that blend the open ocean and ice-covered fluxes in these regions^51,53^. To assess the impact of this uncertainty, a number of sensitivity tests were performed where the ERA5 air−sea heat fluxes in nearby ice-covered regions were merged with those from the COARE bulk flux parameterization to represent the heat flux over the open ocean regions of the marginal ice zone as a function of ice concentration. These tests provided sufficient confidence in the ERA5 air−sea heat fluxes within the marginal ice zone for this study.

## Supplementary information

Supplementary Information

## Data Availability

The ERA5 Reanalysis data used in this paper is available from the Copernicus Climate Data Store at: https://cds.climate.copernicus.eu/.
