# Supplementary material for: Sea-ice retreat suggests re-organization of water mass transformation in the Nordic and Barents Seas
Source: Nat Commun. 2022 Jan 10;13:67. doi: 10.1038/s41467-021-27641-6 (PMC8748645; doi:10.1038/s41467-021-27641-6)
Supplement: Supplementary file 1 — Supplementary Information [file 41467_2021_27641_MOESM1_ESM.pdf]

Supplementary Information for

Evolving air-sea interaction due to Sea-ice retreat suggests re-organisation of water mass transformation in the Nordic and Barents Seas

Authors: G.W.K. Moore[1,2*], K. Våge[3,4], I.A. Renfrew[5] and R.S. Pickart[6]

Nature Communications

[1]Department of Physics, University of Toronto, Toronto, Canada

[2]Department of Chemical and Physical Sciences, University of Toronto Mississauga, Mississauga, Canada

[3]Geophysical Institute, University of Bergen, Bergen, Norway

[4]Bjerknes Centre for Climate Research, Bergen, Norway

[5]School of Environmental Sciences, University of East Anglia, Norwich, UK

[6]Woods Hole Oceanographic Institution, Woods Hole, Massachusetts, USA

[*]Corresponding author: gwk.moore@utoronto.ca

Contents of this file

Supplementary Figures 1-3…………………………………………………………….2-4

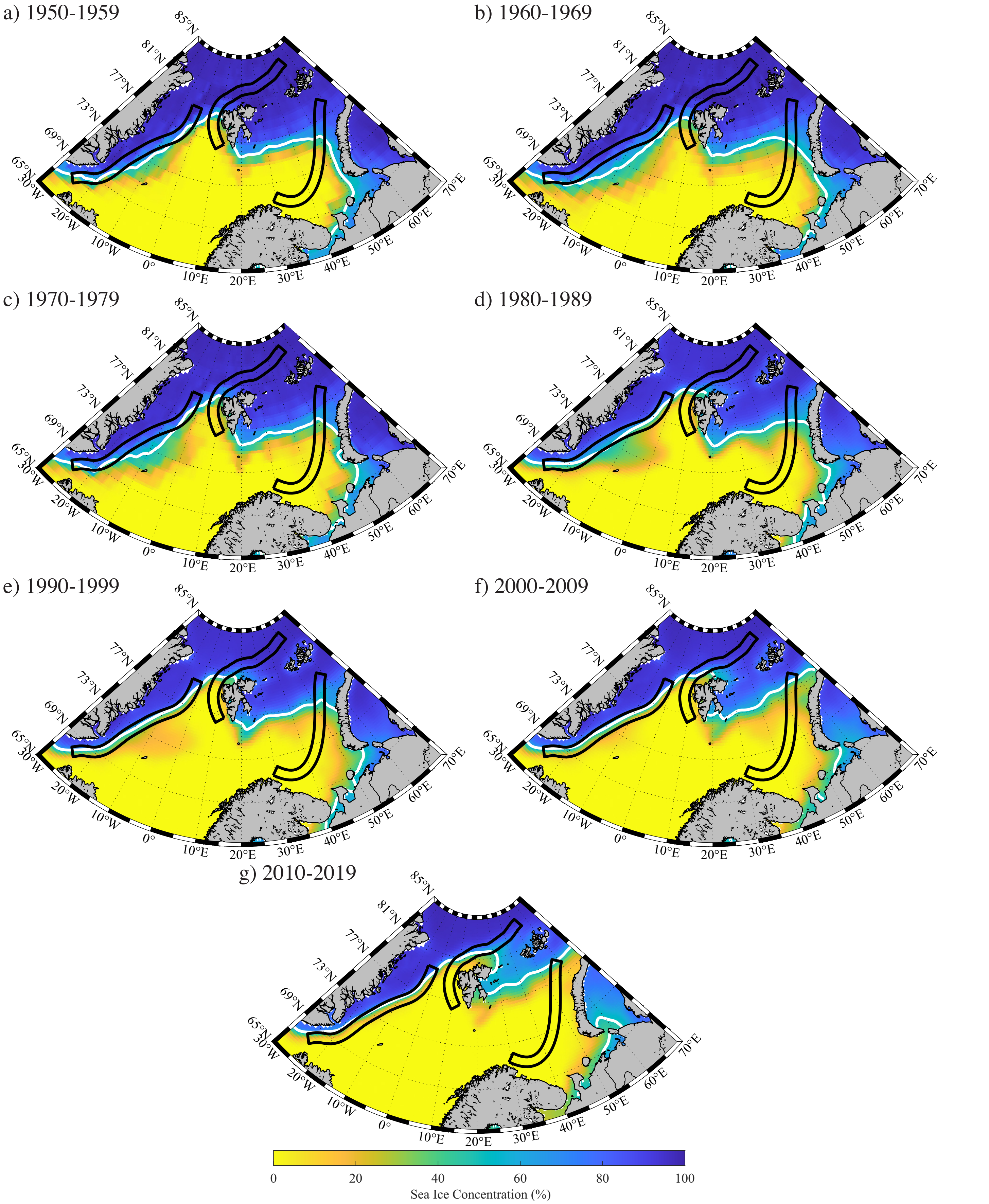

Supplementary Figure 1) Spatiotemporal variability in winter mean ice concentration (%) over the Nordic and Barents Seas from the ERA5 Reanalysis. Decadal means are shown for the period 1950-2020. The domains associated with the EGC, and the SB and BSB of the Atlantic Water Boundary Current are shown in black. The 50% sea ice concentration contour is shown in white.

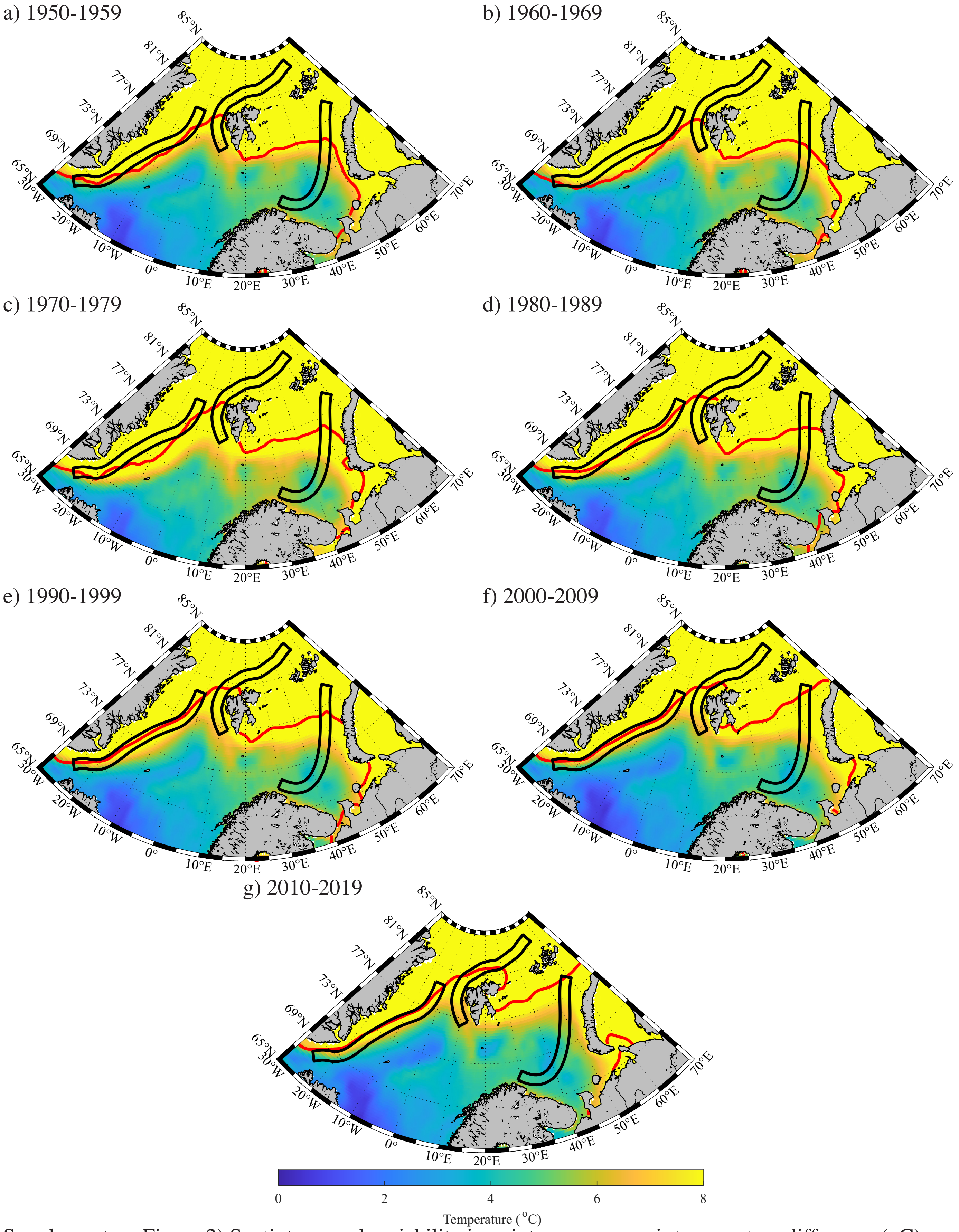

Supplementary Figure 2) Spatiotemporal variability in winter mean sea-air temperature difference (oC) over the Nordic and Barents Seas from the ERA5 Reanalysis. Decadal means are shown for the period 1950-2020. The domains associated with the EGC, and the SB and BSB of the Atlantic Water Boundary Current are shown in black. The 50% sea ice concentration contour is shown in red.

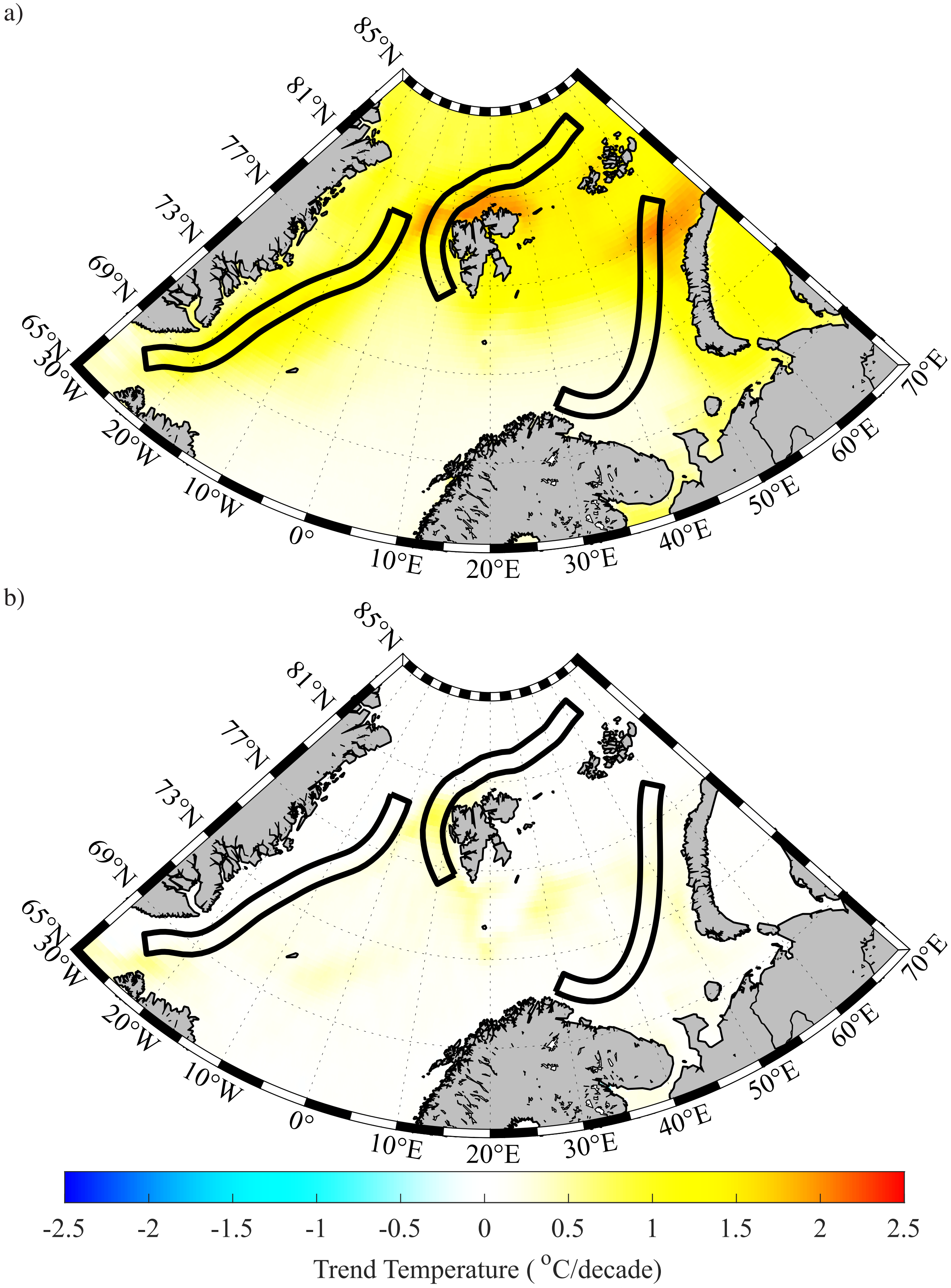

Supplementary Figure3) The trend (ºC/decade) in the ERA5 winter mean: a) 2m Air temperature and b) SST for 1950-2020. The domains associated with the East Greenland Current (EGC) , the Svalbard Branch (SB) and the Barents Sea Branch (BSB) of the Atlantic Water Boundary Current are shown in black.
